# Predictors and clinical implications of residual mitral regurgitation following left ventricular assist device therapy

**DOI:** 10.1136/openhrt-2022-002240

**Published:** 2023-06-14

**Authors:** Harish Sharma, Boyang Liu, Mengshi Yuan, Iqra Shakeel, Andrew Morley-Smith, Alice Hatch, Joseph Bradley, Colin Chue, Saul G Myerson, Richard Paul Steeds, Sern Lim

**Affiliations:** 1 Department of Cardiology, University Hospitals Birmingham NHS Foundation Trust, Birmingham, UK; 2 Institute of Cardiovascular Sciences, University of Birmingham College of Medical and Dental Sciences, Birmingham, UK; 3 Harefield Hospital, Guy's and St Thomas' NHS Foundation Trust, London, UK; 4 Department of Cardiovascular Medicine, University of Oxford, Oxford, UK

**Keywords:** Heart-Assist Devices, HEART FAILURE, Mitral Valve Insufficiency

## Abstract

**Background:**

Correction of mitral regurgitation (MR) at the time of left ventricular assist device (LVAD) implantation remains controversial. There is conflicting evidence regarding the clinical impact of residual MR, and studies have not examined whether MR aetiology or right heart function impacts the likelihood of residual MR.

**Methods:**

This is a retrospective single-centre study of 155 consecutive patients with LVAD implantation from January 2011 to March 2020. Exclusion criteria were no MR pre-LVAD (n=8), inaccessible echocardiography (n=9), duplicate records (n=10) and concomitant mitral valve repair (n=1). Statistical analysis was performed using STATA V.16 and SPSS V.24.

**Results:**

Carpentier IIIb MR aetiology was associated with more severe MR pre-LVAD (severe 18/27 (67%) vs non-severe 32/91 (35%), p=0.004) and a higher likelihood of residual MR (8/11 (72%) vs 30/74 (41%), p=0.045). Of 95 patients with significant MR pre-LVAD, 15 (16%) had persistent significant MR, which was associated with higher mortality (p=0.006), post-LVAD right ventricle (RV) dilatation (10/15 (67%) vs 28/80 (35%), p=0.022) and RV dysfunction (14/15 (93%) vs 35/80 (44%), p<0.001). Aside from ischaemic aetiology, other pre-LVAD parameters that were associated with significant residual MR included left ventricular end-systolic diameter (LVESD) (6.9 cm (5.7–7.2) vs 5.9 cm (5.5–6.5), p=0.043), left atrial volume index (LAVi) (78 mL/m^2^ (56–88) vs 57 mL/m^2^ (47–77), p=0.021), posterior leaflet displacement (2.5 cm (2.3–2.9) vs 2.3 cm (1.9–2.7), p=0.042) and basal right ventricular end-diastolic diameter (RVEDD) (5.1±0.8 cm vs 4.5±0.8 cm, p=0.010).

**Conclusion:**

LVAD therapy improves MR and tricuspid regurgitation severity in the majority, but 14% have persistent significant residual MR, associated with right ventricular dysfunction and higher long-term mortality. This may be predicted pre-LVAD by greater LVESD, RVEDD and LAVi and by ischaemic aetiology.

**WHAT IS ALREADY KNOWN ON THIS TOPIC**

- Left ventricular assist device (LVAD) implantation improves secondary mitral regurgitation (MR); however, the predictors and implications of residual MR are conflicting.

**WHAT THIS STUDY ADDS**

- This study shows that persistent ≥ moderate residual MR after LVAD implantation is associated with greater long-term mortality.
- Echocardiographic predictors include ischaemic aetiology and greater left ventricular end-systolic diameter, right ventricular end-diastolic diameter and left atrial volume index.

**HOW THIS STUDY MIGHT AFFECT RESEARCH, PRACTICE OR POLICY**

- A prospective study is required to evaluate whether patients with the echocardiographic predictors of significant residual MR identified herein should have MR correction before LVAD implantation and whether this improves clinical outcomes.

## Introduction

In patients undergoing left ventricular assist device (LVAD) therapy, the reported prevalence of moderate or severe mitral regurgitation (MR) varies widely between 18% and 77%.^1–3^ Concomitant mitral valve repair may reduce residual MR and pulmonary vascular resistance (PVR), thereby improving right heart function, reducing heart failure (HF) readmission rates^4^ and allowing certain patients with high PVR to become transplant candidates,^5^ but at the cost of increased risk of surgical complications. The impact on mortality is uncertain.^4 6–8^ The grouping together of patients with moderate and severe MR also complicates interpretation of the results.^1 9^ Consequently, there is still a lack of consensus regarding the benefit of concomitant surgical correction of MR during LVAD implantation,^10^ particularly in patients with severe MR. LVAD therapy improves MR^1 11 12^ by unloading the left ventricle (LV), allowing reverse remodelling and correction of subvalvular distortion, but the clinical implication of residual MR is not well understood.

Analysis of the MOMENTUM 3 trial found up to 85% of patients with significant MR pre-LVAD were free of moderate or severe MR at 2 years, with data suggesting 2-year survival rates were unaffected by the presence or severity of MR pre-LVAD and post-LVAD implantation.^9 12^ However, other studies suggested that patients with residual MR may be at risk of poorer outcomes, including further hospitalisation and death.^2 7 13^ In addition, the effect of residual MR on the right ventricle (RV) and tricuspid regurgitation (TR) is unclear, as prior studies have evaluated MR and TR in isolation.^14 15^ Identifying predictors of residual MR following LVAD implant^3^ and the associated changes in RV and TR may guide intervention.

This study had three aims: (1) to describe the aetiology of MR and whether this predicts residual MR; (2) to investigate the relationship between residual MR and right heart size and function and the probability of pulmonary hypertension (PH); and (3) to determine the impact of residual MR on outcome.

## Materials and methods

### Patient selection

The Queen Elizabeth Hospital Birmingham (QEHB) is one of six advanced HF centres in the UK. A prospectively maintained database of patients undergoing LVAD implantation at the QEHB was retrospectively searched. Inclusion criteria included all patients undergoing LVAD implantation with MR on pre-LVAD echocardiography and follow-up echocardiography. Exclusion criteria included no MR pre-LVAD (n=8), inaccessible pre-LVAD echocardiography (n=9), duplications arising due to LVAD exchange (n=10) and concomitant mitral valve surgery (n=1). Data pertaining to age, comorbidities, blood results and echocardiographic reports were obtained from hospital records between January 2011 and March 2020. HF admissions were defined by discharge letters and mortality was determined from hospital records, which are linked to data from the Office for National Statistics. Patient and public planning and involvement in this study were not possible due to its retrospective design.

### Echocardiography

Transthoracic or transoesophageal echocardiography was performed using EPIQ machines (Philips Medical Systems, Amsterdam, The Netherlands). Scans were performed on clinical grounds and reported by accredited members of the British Society of Echocardiography (BSE). Where possible, the BSE minimum data set was acquired^16^ and MR grade adjudged according to the multiparametric approach recommended by international guidelines.^17–19^ In the case of multiple echocardiograms, the scan immediately prior to LVAD implantation was analysed as the pre-LVAD echocardiogram, and the latest post-LVAD scan was analysed as the follow-up scan. LV diameters and vena contracta were assessed in the parasternal long-axis (PLAX) view. Proximal isovelocity surface area, effective regurgitant orifice area and regurgitant volume were derived from the proximal flow convergence method assessed in the apical four-chamber view. Left atrial and left ventricular volumes were assessed using the Simpson’s biplane method and indexed according to the Mosteller calculation of body surface area. Mitral tethering height and area were calculated offline in PLAX view in mid-systole. Tethering height was defined as the maximum perpendicular distance to the coaptation point from the annular plane. Tethering area was defined as the area enclosed by the mitral annular plane and mitral leaflets. Mitral annular diameters and interpapillary distances were measured in the parasternal short axis. The mechanism of MR was determined according to the Carpentier classification by two blinded senior echocardiographers (AH and JB).

Right ventricular end-diastolic diameter (RVEDD) was measured in the four-chamber view at the basal level. RV function was assessed by the RV fractional area change (FAC) using a threshold of >35%, tricuspid annular plane systolic excursion (TAPSE) method (normal >17 mm), or by subjective visual assessment if FAC or TAPSE was not measurable. Classification of PH was performed in accordance with recent guidelines.^20^ Ramp studies were performed to optimise LVAD settings prior to discharge and at 3 months postdischarge.

### Medical and interventional therapy

LVADs are commissioned as bridge to candidacy or transplantation in the UK. Therefore, none of the patients in this study underwent LVAD implant as destination therapy. All patients treated by LVAD therapy were seen in the advanced HF clinic for optimisation of HF medications according to guideline recommendations prior to consideration of LVAD therapy. In accordance with the UK heart allocation scheme, patients were placed under the non-urgent category following LVAD implant and only prioritised to the urgent category in the presence of significant LVAD-related complications (eg, systemic infection or thrombosis). As a result, all but one patient receiving cardiac transplantation did so due to LVAD-related complications. HeartMate II (Abbott, Illinois, USA) was the only device used up to October 2015 and HeartMate III devices were used in all implants after October 2015. We do not routinely repair or replace mitral and tricuspid valve at the time of LVAD implant. Only one patient (HeartMate II) underwent concomitant mitral valve surgery early in our LVAD experience.

### Statistical analysis

Continuous variables are expressed as mean±SD or median with IQR for variables following a non-normal distribution. Normally and non-normally distributed continuous variables were compared using Student’s two-tailed t-test or Mann-Whitney U test, respectively, depending on the degree of normality as judged by the Shapiro-Wilk test. Qualitative data were assessed using Fisher’s exact text. Time-to-event analysis assessed survival using Kaplan-Meier plots. Independent predictors of mortality were assessed by a multivariable stepwise Cox regression model. A p value of <0.05 was considered statistically significant. Statistical analysis was performed using SPSS V.26 and STATA V.16.

### Data analysis

Change in MR and TR severity was compared from pre-LVAD to the latest follow-up echocardiography, after excluding patients who did not have follow-up echocardiography (eg, died before follow-up echocardiography could be performed). Patients were excluded from outcome analysis after receiving cardiac transplantation. Significant residual MR and TR were defined as moderate or severe regurgitation on the latest echocardiography.

## Results

### Baseline characteristics

**Figure 1:**
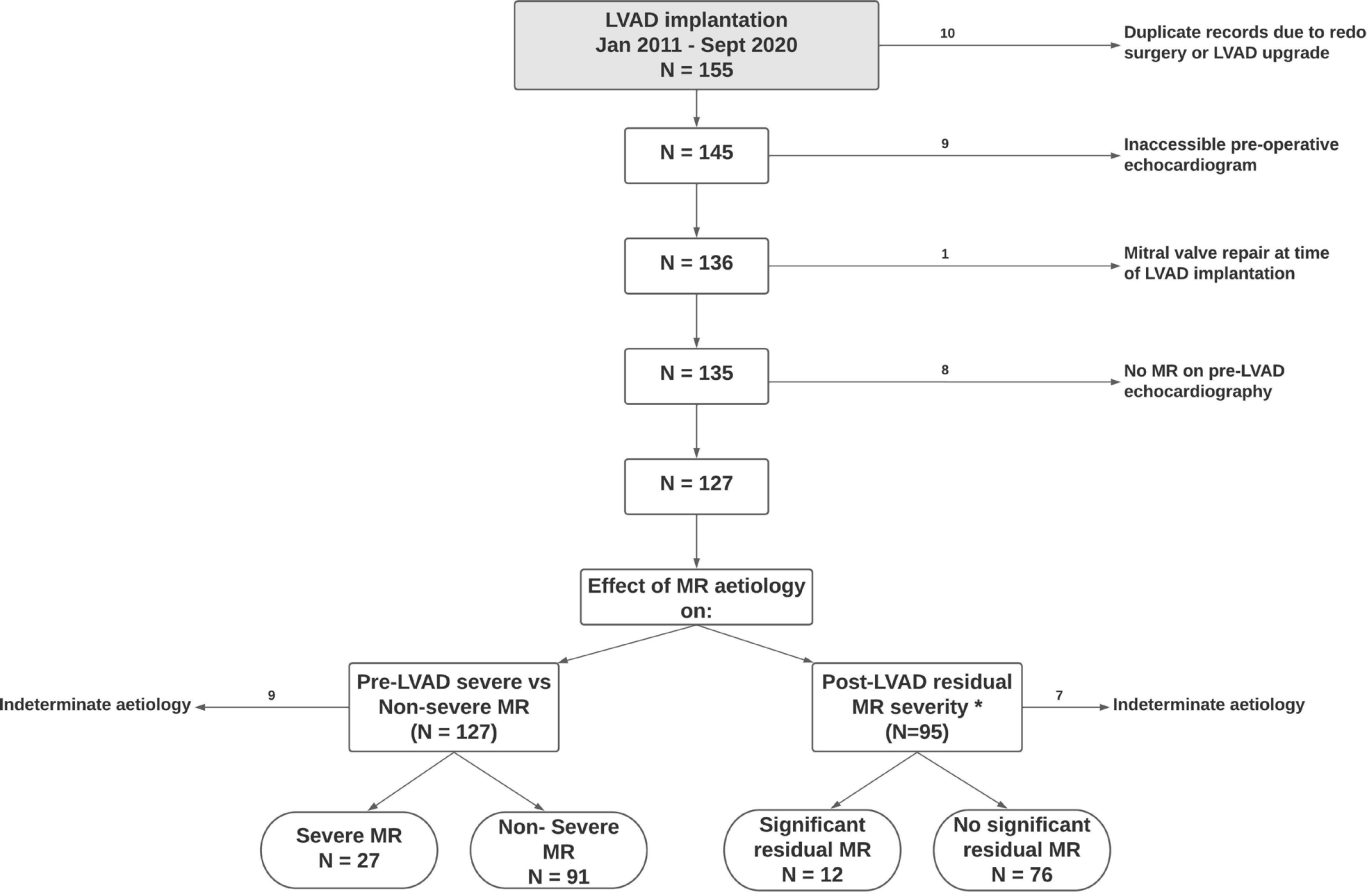
Flow chart showing the selection and exclusion of patients. *Analysis of patients with residual MR excluded those with mild MR pre-LVAD and those who died or underwent cardiac transplantation before follow-up echocardiography could occur. Significant MR was defined as moderate or severe regurgitation. LVAD, left ventricular assist device; MR, mitral regurgitation.

A total of 155 patients had LVAD implantation between January 2011 and March 2020. Twenty-eight patients were excluded due to duplicate records (n=10), inaccessible pre-LVAD echocardiography (n=9), mitral valve repair (n=1) or no MR pre-LVAD (n=8) (figure 1). All remaining 127 included patients who had MR (mild 27 (21%), moderate 75 (55%), severe 29 (23%)). Patients had a mean age of 55±8 years and 107 of 127 (84%) were men. The mechanism of MR by Carpentier classification was pure type I in 58 (46%), pure type IIIb in 50 (39%), mixed I/IIIb in 7 (6%) and indeterminate in 9 (7%). A total of 71 out of 127 (56%) patients had implanted defibrillator devices. Baseline cohort characteristics are shown in table 1.

**Table 1:** Baseline characteristics of the cohort pre-LVAD implantation

Parameter	Value
Age (years)	55±8
Male sex, n (%)	107 (84)
BMI (kg/m^2^)	27±4
NT-proBNP (ng/L)	5040 (2706–7730)
eGFR (mL/min/1.73 m^2^)	55±20
CRT-D/ICD, n (%)	71 (56)
INTERMACS profile, n (%)	
INTERMACS 1	10 (8)
INTERMACS 2	34 (27)
INTERMACS 3	83 (65)
Mechanical circulatory support, n (%)	
IABP	12 (9)
ECMO	7 (6)
Impella	8 (6)
Concomitant procedure required and performed, n (%)	
ASD/PFO closure	6 (5)
Bioprosthetic AVR	6 (5)
Number of days pre-LVAD echocardiography performed	20 (8–49)
TTE, n (%)	119 (94)
LVEDD (cm)	6.6±0.9
LVESD (cm)	5.9±1.0
LVEDVi (mL/m^2^)	122±50
LVESVi (mL/m^2^)	99±57
LVEF (%)	17±8
LAVi (mL/m^2^)	60±32
Mechanism of MR, n (%)	
Annular dilatation (Carpentier type I)	58 (46)
Ischaemic (Carpentier type IIIb)	50 (39)
Mixed aetiology (ischaemic and annular dilatation)	8 (6)
Indeterminable aetiology	11 (7)

Values represent mean±SD, median (IQR) or proportion of patients (%).

NT-proBNP levels shown are values closest to the time of LVAD implantation.

ASD, atrial septal defect; AVR, aortic valve replacement; BMI, body mass index; CRT-D, cardiac resynchronisation therapy defibrillator; ECMO, extracorporeal membrane oxygenation; eGFR, estimated glomerular filtration rate; IABP, intra-aortic balloon pump; ICD, implantable cardiac defibrillator; INTERMACS, Interagency Registry for Mechanically Assisted Circulatory Support; LAVi, indexed left atrial volume; LVAD, left ventricular assist device; LVEDD, left ventricular end-diastolic diameter; LVEDVi, indexed left ventricular end-diastolic volume; LVEF, left ventricular ejection fraction; LVESD, left ventricular end-systolic diameter; LVESVi, indexed left ventricular end-systolic volume; MR, mitral regurgitation; NT-proBNP, N-terminal B-type natriuretic peptide; PFO, patent foramen ovale; TTE, transthoracic echocardiography.

### Course of mitral and tricuspid regurgitation with LVAD therapy

**Figure 2:**
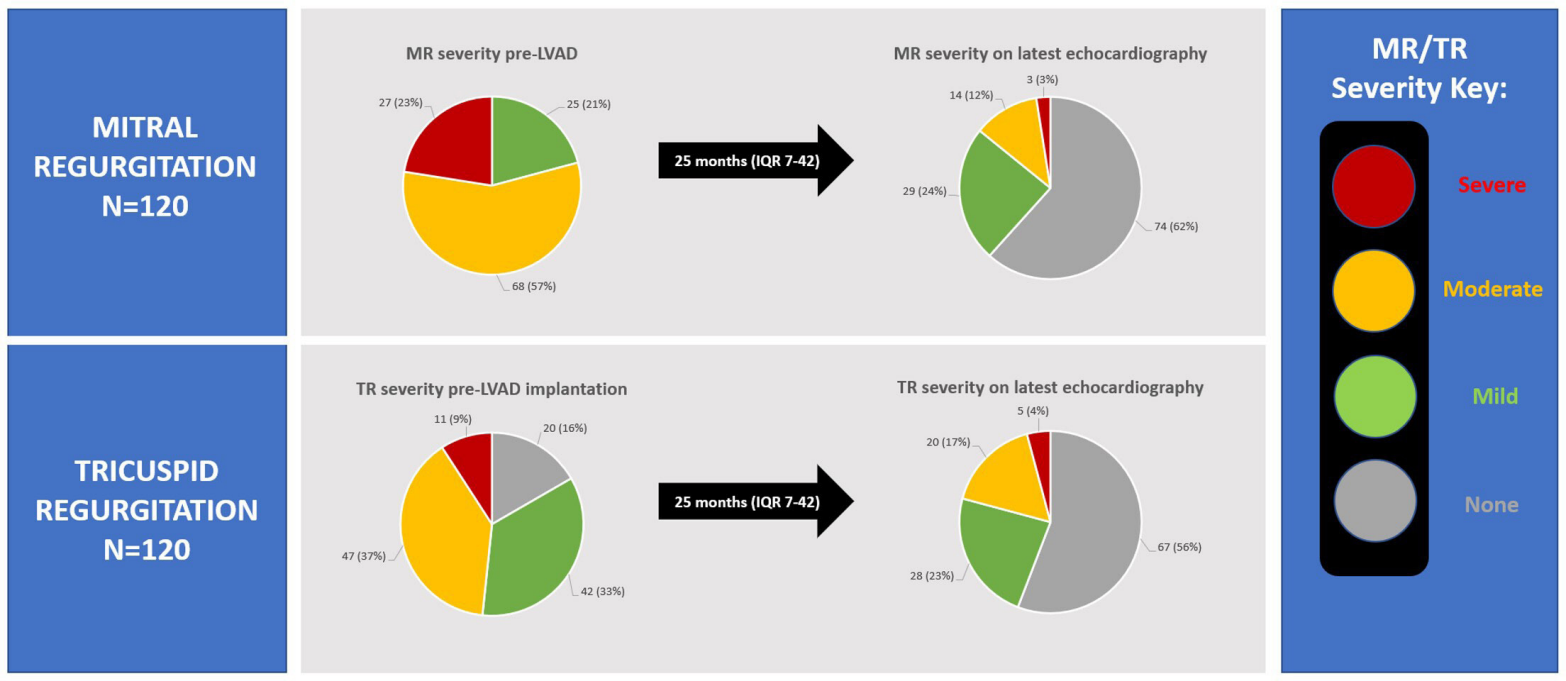
Pie charts demonstrating change in mitral and tricuspid regurgitation severity on echocardiography from pre-LVAD implantation to latest follow-up scan (n=120). LVAD, left ventricular assist device; MR, mitral regurgitation; TR, tricuspid regurgitation.

A follow-up echocardiogram was recorded in 120 patients after a median of 25 months (IQR 7–42). Seven patients died before follow-up echocardiography could be performed. The severity of MR improved significantly with LVAD therapy, such that only 14% of patients had moderate or severe residual MR at latest follow-up, compared with 79% before LVAD implantation. The severity of TR also improved; 21% of patients had moderate or severe residual TR after LVAD implantation, compared with 48% pre-LVAD (figure 2). None of the patients without MR pre-LVAD developed significant MR on follow-up echocardiography.

### Effect of MR aetiology on pre-LVAD MR severity

Twenty-nine patients had severe MR at baseline compared with 98 patients without severe MR pre-LVAD. The characteristics of these patients are shown in table 2. Excluding the nine patients with indeterminate aetiology (cohort size: severe MR=27, non-severe MR=91), patients with severe MR were more likely to have purely ischaemic (Carpentier type IIIb) aetiology of MR (18/27 (67%) vs 32/91 (35%), p=0.004), while patients with non-severe MR were more likely to have MR secondary to annular dilatation (Carpentier type I) (50/91 (55%) vs 8/27 (30%), p=0.021). Echocardiography, right heart catheter and procedural characteristics of these cohorts are shown in online supplemental tables 1–3.

10.1136/openhrt-2022-002240.supp1 Supplementary data

**Table 2:** Echocardiographic characteristics of patients with and without severe MR pre-LVAD

	n	Severe MR (n=29)	n	Non-severe MR (n=98)	P value
E wave velocity (cm/s)	26	113 (104–136)	93	97 (82–118)	<0.001*
A wave velocity (cm/s)	19	42 (30–54)	54	35 (30–56)	0.767
E:A ratio	19	2.7 (2.2–3.8)	53	2.6 (1.9–3.3)	0.362
PISA (cm)	26	0.86 (0.80–1.0)	76	0.60 (0.5–0.7)	<0.001*
VC (cm)	19	0.79±0.32	63	0.57±0.18	<0.001*
EROA (cm^2^)	24	0.44 (0.37–0.50)	68	0.20 (0.15–0.28)	<0.001*
RVol (mL)	23	47±26	65	24±12	<0.001*
RVol/EDV	23	0.18 (0.13–0.22)	64	0.08 (0.06–0.15)	<0.001*
RF (%)	20	54±15	64	37±13	<0.001*
Anterior leaflet displacement (Da) (cm)	23	3.0 (2.5–4.2)	91	2.7 (2.2–3.7)	0.170
Posterior leaflet displacement (Dp) (cm)	23	2.6±0.6	91	2.1±0.5	<0.001*
Tethering proportion (Da:Dp)	23	1.4 (1.1–1.5)	91	1.4 (1.1–1.6)	0.304
Tethering area (cm^2^)	23	5.0 (4.0–6.6)	91	3.7 (2.9–4.8)	0.002*
Tethering height (cm)	23	1.9 (1.6–2.0)	91	1.5 (1.3–1.8)	0.011*
Interpapillary distance diastole (cm)	15	3.2±0.8	53	3.2±0.7	1.0
Interpapillary distance systole (cm)	15	2.4±0.9	53	2.6±0.7	0.363
Annular diameter: anteroposterior (cm)	18	2.2±0.5	74	2.2±0.5	1.0
Annular diameter: intercommissural (cm)	18	3.9±0.5	73	3.9±0.7	1.0
TAPSE (mm)	25	15±4	92	16±5	0.358

*Statistical significance

EROA, effective regurgitant orifice area; LVAD, left ventricular assist device; MR, mitral regurgitation; PISA, proximal isovelocity surface area; RF, regurgitant fraction; RVol, regurgitant volume; RVol/EDV, indexed regurgitant volume/left ventricular end-diastolic volume; TAPSE, tricuspid annular plane systolic exclusion; VC, vena contracta.

### Outcomes in patients with and without severe MR pre-LVAD

**Figure 3:**
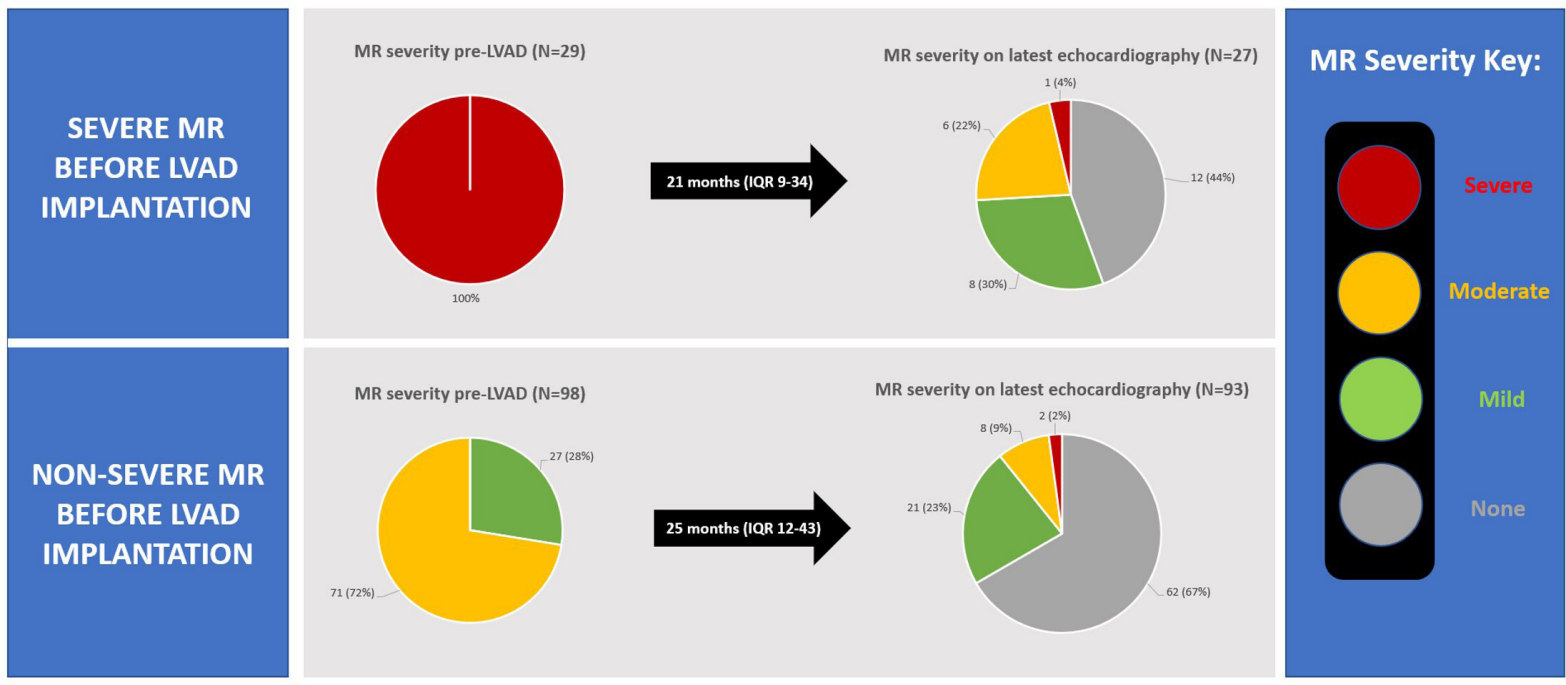
Pie charts demonstrating change in MR severity among patients with severe MR pre-LVAD and non-severe MR pre-LVAD (left). The pie charts on the right demonstrate the relative severities of MR on latest echocardiography. LVAD, left ventricular assist device; MR, mitral regurgitation.

**Figure 4:**
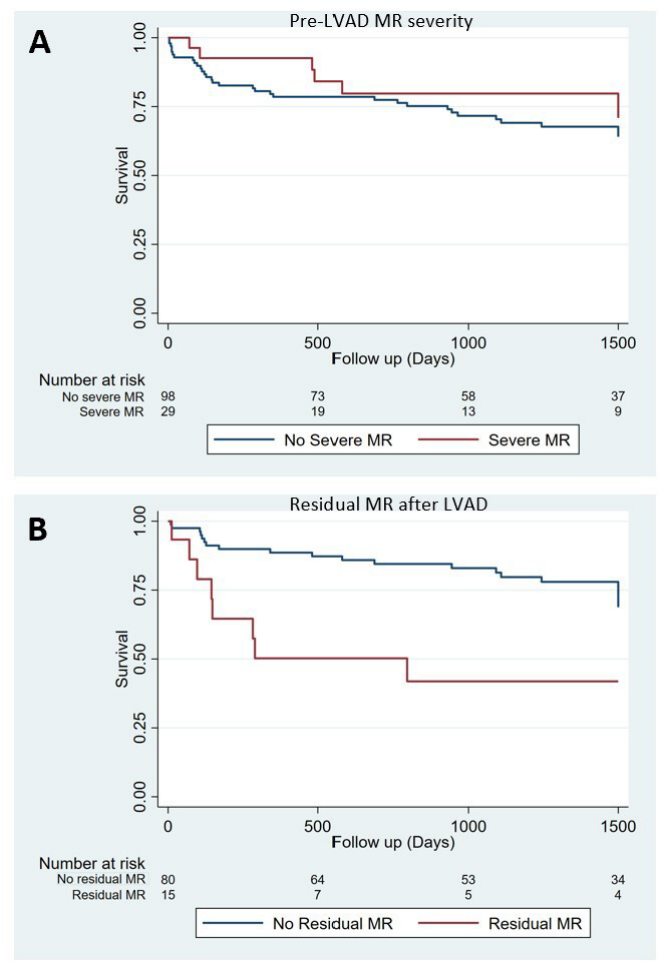
Kaplan-Meier plots of survival free from heart transplantation. (A) Patients with baseline severe MR (n=98; red line) versus no severe MR (n=29; blue line). (B) Patients with (n=15; red line) and without (n=80; blue line) significant residual MR among patients with moderate or severe MR pre-LVAD. LVAD, left ventricular assist device; MR, mitral regurgitation.

In patients with severe MR pre-LVAD, follow-up transthoracic echocardiography was performed in 27 out of 29 patients, with a median follow-up of 21 months (IQR 9–34). A demonstrable improvement in MR severity (by >1 grade) was observed in 26 out of 27 (96%) patients (figure 3). After a median of 5 years (IQR: 4–6) post-LVAD implantation, there were no significant differences in HF hospitalisation (6/24 (25%) vs 21/91 (23%), p=0.843) or death (6/24 (21%) vs 34/91 (33%), p=0.258) between patients with and without severe MR pre-LVAD (figure 4A and online supplemental table 4).

### Predicting patients with residual MR and association with MR aetiology

**Figure 5:**
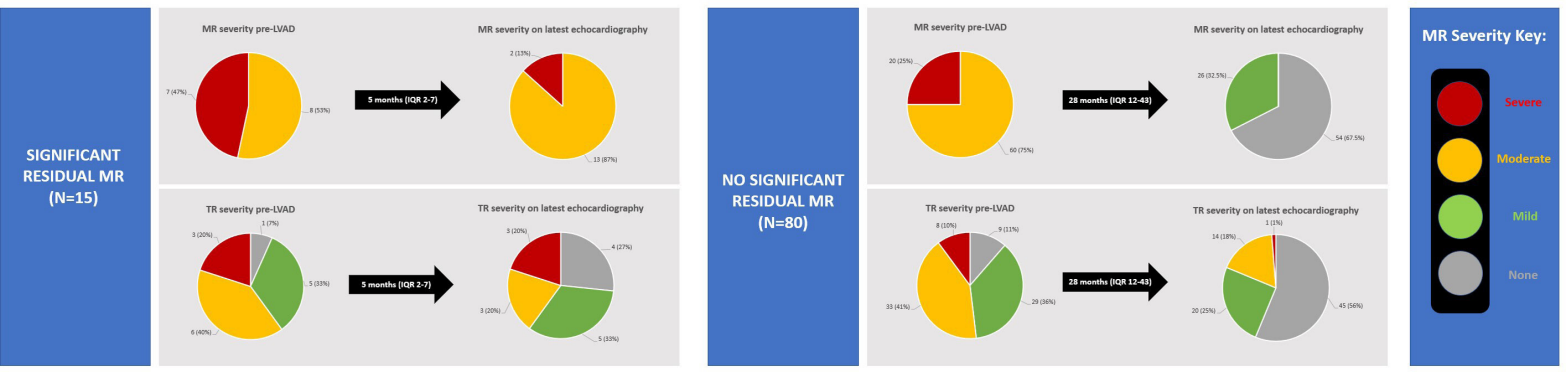
Pie charts showing the severity of MR and TR in patients with significant residual MR after LVAD implantation. Significant residual MR was defined as ≥ moderate severity. This analysis included only patients with ≥ moderate MR at baseline who had follow-up echocardiography. LVAD, left ventricular assist device; MR, mitral regurgitation; TR, tricuspid regurgitation.

A total of 100 patients had significant (≥ moderate) MR pre-LVAD, among whom 95 had follow-up echocardiography. Fifteen patients had significant (≥ moderate) residual MR, while 80 patients had no significant residual MR (figure 5). Patients with and without significant residual MR were of similar age (58±9 years vs 55±9 years, p=0.239) and had similar baseline estimated glomerular filtration rate (eGFR) (44 mL/min/1.73 m^2^ (IQR 35–53) vs 59 mL/min/1.73 m^2^ (IQR 42–73), p=0.066) and levels of N-terminal B-type natriuretic peptide (6706 ng/L (IQR 5941–7586) vs 4914 ng/L (IQR 2548–8914), p=0.481). Furthermore, there were no differences in LVAD speed settings (6440±1555 rpm vs 6522±1678 rpm, p=0.861), indexed flow (2.8±0.5 L/min/m^2^ vs 2.7±0.5 L/min/m^2^, p=0.479) or LVAD complications (13% vs 20%, p=0.545) (online supplemental table 5).

Patients with significant residual MR were more likely to have type IIIb (restrictive tethering) MR aetiology pre-LVAD (9/12 (75%) vs 31/76 (41%), p=0.027), where aetiology was able to be determined. Conversely, patients without significant residual MR were more likely to have purely type I lesions (annular dilatation) pre-LVAD (40/76 (53%) vs 2/12 (17%), p=0.020). Patients with residual MR had greater left ventricular end-systolic diameter (LVESD) in systole, larger left atrial volume index (LAVi) and basal RVEDD, and greater posterior leaflet displacement prior to LVAD implantation (table 3). Furthermore, significant residual MR was associated with worse cardiac output on pre-LVAD right heart catheterisation (RHC) (2.8±0.9 L/min vs 3.4±0.8 L/min, p=0.045).

**Table 3:** Preoperative echocardiographic predictors of significant residual MR after LVAD therapy in patients with baseline moderate or severe MR

	n	Significant MR post-LVAD	n	No significant MR post-LVAD	P value
Number of days pre-LVAD	15	23 (15–48)	80	22 (9–43)	0.744
LVEDD (cm)	15	7.3 (6.3–7.8)	78	6.5 (6.1–7.2)	0.055
LVESD (cm)	15	6.9 (5.7–7.2)	77	5.9 (5.5–6.5)	0.043*
LVEDVi (mL/m^2^)	15	151 (102–172)	77	114 (95–146)	0.072
LVESVi (mL/m^2^)	15	106 (78–131)	77	92 (72–113)	0.190
LVEF (%)	15	13 (9–20)	80	15 (12–22)	0.146
LAVi (mL/m^2^)	14	78 (56–88)	74	57 (47–77)	0.029*
E wave (cm/s)	13	114±45	75	105±23	0.273
Anterior leaflet displacement (Da) (cm)	13	3.0 (2.5–3.9)	73	3.0 (2.2–4.1)	0.819
Posterior leaflet displacement (Dp) (cm)	13	2.5 (2.3–2.9)	73	2.3 (1.9–2.7)	0.021*
Tethering proportion (Da:Dp)	13	1.4 (1.0–1.5)	73	1.4 (1.1–1.6)	0.201
Tethering area (cm^2^)	13	4.7 (3.3–6.9)	73	4.1 (3.5–5.1)	0.286
Tethering height (cm)	13	1.7 (1.2–2.0)	73	1.7 (1.4–1.9)	0.928
Interpapillary distance (diastole) (cm)	11	2.9 (2.3–4.0)	41	3.2 (2.8–3.7)	0.439
Interpapillary distance (systole) (cm)	11	2.2 (1.6–3.3)	42	2.4 (2.1–3.1)	0.398
Annular diameter (AP) (cm)	11	2.2±0.5	64	2.2±0.5	1.0
Annular diameter (ALPM) (cm)	11	4.0 (3.8–4.1)	63	3.8 (3.6–4.3)	0.311
Basal LV:RV ratio	12	1.4 (1.2–1.5)	73	1.4 (1.3–1.6)	0.325
TR Vmax	13	3.2 (3.0–3.6)	71	3.1 (2.6–3.4)	0.345
High likelihood of pHTN	13	8 (53)	73	43 (59)	0.859
RVEDD (cm)	15	5.1±0.8	72	4.5±0.8	0.010*
TAPSE (mm)	14	14.6±3.9	74	15.8±5.1	0.407
Carpentier mechanism					
Type I only	12	2 (17)	76	40 (53)	0.020*
Type IIIb only	12	9 (75)	76	31 (41)	0.027*
Mixed	12	1 (8)	76	5 (7)	0.823

Values represent mean±SD, median (IQR) or proportion of patients (%).

ALPM, anterolateral to posteromedial; AP, anteroposterior; LAVi, left atrial volume index; LV, left ventricular; LVAD, left ventricular assist device; LVEDD, left ventricular end-diastolic diameter; LVEDVi, left ventricular end-diastolic volume index; LVEF, left ventricular ejection fraction; LVESD, left ventricular end-systolic diameter; LVESVi, left ventricular end-diastolic volume index; MR, mitral regurgitation; pHTN, pulmonary hypertension; RV, right ventricular; RVEDD, right ventricular end-diastolic diameter; TAPSE, tricuspid annular plane systolic excursion; TR, tricuspid regurgitation; Vmax, maximal velocity.

Compared with pre-LVAD echocardiography, on follow-up scans, patients with significant residual MR lacked significant LV reverse remodelling when comparing LVEDD (significant residual MR: 7.2±1.0 cm vs 6.6±0.7 cm, p=0.09; no significant residual MR: 6.6±0.7 cm vs 5.7±0.9 cm, p<0.001).

### Clinical implication of residual MR and association with the right heart

Among patients with significant persistent (residual) MR after LVAD therapy, the prevalence of severe TR was unchanged at follow-up compared with pre-LVAD (20%). By comparison, in patients without significant residual MR, rates of severe TR improved from 10% at baseline to only 1% at follow-up (figure 5).

At follow-up, significant residual MR was associated with a higher prevalence of RV dilatation (10/15 (67%) vs 28/80 (35%), p=0.022) and reduced RV function (14/15 (93%) vs 35/80 (44%), p<0.001). However, the proportion of patients with high likelihood of PH on echocardiography did not differ between the groups (5/15 (33%) vs 24/80 (30%), p=0.797). Moreover, the groups did not differ with respect to mean pre-LVAD pulmonary arterial pressure (40±7 mm Hg vs 40±10 mm Hg, p=0.944), pulmonary capillary wedge pressure (28±6 mm Hg vs 27±6 mm Hg, p=0.582) or transpulmonary pulmonary gradient (12±5 mm Hg vs 13±6 mm Hg, p=0.573) on RHC.

After a median follow-up of 5 years (IQR: 4–7), patients with significant residual MR had a higher all-cause mortality than patients without significant residual MR (log-rank test of survival free from heart transplantation p=0.006) (figure 4B). A multivariable stepwise regression analysis was performed comparing residual MR with multiple preoperative risk factors, including age, eGFR, diabetes mellitus, left ventricular ejection fraction, INTERMACS (Interagency Registry for Mechanically Assisted Circulatory Support) profile, cardiac output on RHC and RVEDD. This found that residual MR is an independent risk factor for mortality (p=0.015, 95% CI 0.134 to 0.804). Rates of HF hospitalisation in non-transplanted individuals did not differ significantly between those with and without residual MR (4/14 (29%) vs 17/72 (24%), p=0.901).

### Limitations

The authors acknowledge the study data are limited by the single-centre, retrospective design. The study is also limited by the small number of patients with severe MR pre-LVAD despite the overall large cohort size. Survival analysis comparing patients with and without severe MR was potentially confounded by the bias of transplantation, which occurred more frequently and on average occurred earlier after LVAD implantation in the severe MR group than in the non-severe MR group. Other confounding factors have been addressed using multivariable regression analysis. However, although there were no patients with severe aortic regurgitation (AR) in the cohort, we do not have data on whether patients had moderate AR. Furthermore, although baseline RHC data were collected, serial measurements and their relationship to the outcome were not studied.

## Discussion

In this study, we found that severe MR was present in more than one in five patients requiring LVAD therapy. Severe MR was most often associated with a Carpentier type IIIb (ischaemic) rather than type I (dilated annulus) mechanism. We found that outcomes were not affected by the severity of MR pre-LVAD, but instead related to the severity of residual MR. At a median of 2 years after LVAD implantation, severe residual MR was observed in less than 3% of patients, but moderate or severe residual MR was observed in 14% of all patients (and 16% of all patients with moderate or severe MR at baseline), consistent with data from the MOMENTUM 3 trial.^12^ Most of these patients had persistent unresolved MR, but in some mild MR had progressed over time. When these two groups are analysed together, the presence of significant residual MR detected on echocardiography at a median of 6 months post-LVAD implantation did not impact the median 5-year mortality (online supplemental table 6). However, in a subgroup analysis of patients with only baseline moderate or severe MR, the presence of significant residual MR at a median of 5 months was associated with a significantly higher risk of a median 5-year mortality. Additionally, patients with significant residual MR had a higher prevalence of right heart dilatation and systolic dysfunction, which may have contributed to the poorer clinical outcomes. Echocardiographic factors which could predict patients *pre-LVAD* who are at risk of persistent MR post-LVAD included those with purely Carpentier type IIIb mechanism, significant posterior leaflet displacement, larger LAVi and greater LVESD.

These results highlight that patients with baseline severe LV dilatation and ischaemic MR aetiology are at risk of significant residual MR after LVAD implantation. While LVAD treatment encourages reverse remodelling, previous studies in patients undergoing mitral valve surgery have shown that reverse remodelling may be less effective in the presence of significant preintervention LV dilatation, resulting in poorer postintervention LV function and outcomes.^21–23^ It is notable that, although patients with and without significant residual MR had pre-LVAD LV systolic diameters in the severe reference range,^24^ the median absolute values of patients with significant residual MR were 10 mm larger. In keeping with this, our study found that unlike their counterparts without significant residual MR, those with significant residual MR failed to have significant LV reverse remodelling. However, it is not clear whether this is the cause or effect of the residual MR. Post-LVAD RV dysfunction may impede effective LVAD unloading of the LV. In our study, patients with residual MR were more likely to have pre-LVAD RV dilatation and post-LVAD RV dilatation and dysfunction and TR. All our patients underwent ramp studies to optimise pump speed setting. Our finding that pump speeds were comparable despite the presence of persistent LV dilatation and residual MR suggests that further unloading may have been limited by RV dysfunction.

The mechanism of MR may also contribute to residual MR in addition to the degree of LV unloading by the LVAD. In those with an ischaemic mechanism, residual MR may persist after LVAD therapy because reverse remodelling of the ventricle does not fully compensate for the tethered and restricted motion of the valve leaflets. The underlying ischaemic changes may also be associated with an elevated end-diastolic pressure–volume relation that may have resulted in higher LV end-diastolic pressure at a given end-diastolic volume. Further work with larger data sets and use of other imaging modalities such as cardiovascular magnetic resonance pre-LVAD implant would be helpful to examine whether the extent of LV fibrosis or strain can predict whether MR of ischaemic aetiology is likely to persist, particularly as MR due to this mechanism is more challenging to repair.

In conclusion, LVAD therapy markedly improves MR in most patients; however, 14% of patients have significant residual MR, which is associated with a higher risk of long-term mortality. These patients may be identified on pre-LVAD echocardiography by larger LVESD, LAVi, posterior leaflet displacement, ischaemic aetiology and RV dilatation. Further studies need to focus on whether pre-LVAD repair is indicated in this cohort.

## Data Availability

Data are available upon reasonable request.
