# Supplementary material for: Predictors and clinical implications of residual mitral regurgitation following left ventricular assist device therapy
Source: Open Heart. 2023 Jun 14;10(1):e002240. doi: 10.1136/openhrt-2022-002240 (PMC10277521; doi:10.1136/openhrt-2022-002240)
Supplement: Supplementary data [file openhrt-2022-002240supp001.pdf]

Table of Contents

1. **Supplementary Table 1……………………………………………………………………….Page 2**
2. **Supplementary Table 2……………………………………………………………………….Page 3**
3. **Supplementary Table 3……………………………………………………………………….Page 4**
4. **Supplementary Table 4……………………………………………………………………….Page 5**
5. **Supplementary Table 5……………………………………………………………………….Page 6**
6. **Supplementary Table 6……………………………………………………………………….Page 7**

Sharma H, *et al. Open Heart* 2023; 10:e002240. doi: 10.1136/openhrt-2022-002240

Supplementary Table 1: Echocardiographic characteristics pre-LVAD

	Severe MR (N = 29)	Non-severe MR (N = 98)	P value
**No. days pre-LVAD**	36 ± 36	38 ± 50	0.842
**TTE (N, %)**	27 (93%)	92 (94%)	0.880
**LVEDD (cm)**	7.0 ± 0.8	6.5 ± 0.9	0.008 **
**LVESD (cm)**	6.1 ± 1.1	5.9 ± 1.0	0.357
**LVEDVi (ml/m^2)**	133 ± 44	118 ± 50	0.148
**LVESVi (ml/m^2)**	107 ± 41	96 ± 59	0.350
**LVEF (%)**	17 ± 9	17 ± 7	1.000
**LAVi (ml/m^2)**	71 ± 29	56 ± 31	0.022 **
**Basal LV/RV ratio**	1.5 ± 0.5	1.4 ± 0.2	0.114
**RVID Base (cm)**	4.4 ± 1.1	4.4 ± 0.8	1.000
**RV FAC (%)**	31 ± 9	23 ± 12	0.001 **
**TAPSE (mm)**	15 ± 4	16 ± 5	0.326
**Impaired RV function (FAC/TAPSE below normal)**	17 (59%)	73 (74%)	0.099
**Significant TR**	19 (66%)	40 (41%)	0.033
**TR Vmax**	3.2 (2.9-3.6)	3.0 (2.6-3.5)	0.120

Supplementary Table 1: Echocardiographic characteristics of patients with and without severe MR pre-LVAD. Values represent mean ± standard deviation or median (interquartile range). MR = mitral regurgitation; TTE = Transthoracic echocardiogram; TOE = Transoesophageal echocardiogram; LVEDD = Left ventricular end-diastolic diameter; LVESD = Left ventricular end-systolic diameter; LVEDVi = Indexed left ventricular end-diastolic volume; LVESVi = Indexed left ventricular end-diastolic volume; RV = Right ventricle; RVID = Right ventricular internal diameter; FAC = Fractional area change; TAPSE = Tricuspid annular plane systolic excursion; TR Vmax = Tricuspid regurgitation maximal velocity - only patients with measurable TR on continuous wave doppler were included (severe MR = 26; non-severe MR = 83)

Supplementary Table 2: Cardiopulmonary exercise and invasive pressure measurements of patients with and without severe MR pre-LVAD implantation

	Severe MR (N = 29)	Non-severe MR (N = 98)	P value
**CARDIOPULMONARY EXERCISE MEASUREMENTS**			
**Days before LVAD**	65 (23-128)	73 (29-127)	0.789
**Max METS**	3.3 (2.3-5)	3.4 (2.5-4.5)	0.874
**VO_2 (ml/kg/min)**	9.2 (7.9-12.2)	8.8 (6.8-11.6)	0.829
**O_2 Pulse (VO_2/HR) (ml/beat)**	8.8 (6.7-10)	8.2 (5.5-9.9)	0.385
**VE/VCO_2 slope**	49 (41-53)	46 (42-51)	0.483
**INVASIVE PRESSURE MEASUREMENTS**			
**Cardiac output (L)**	3.2 (2.8-3.5)	3.6 (2.8-3.9)	0.142
**Mean PA pressure (mmHg)**	40 $\pm$ 13	40 $\pm$ 9	1.0
**PCWP (mmHg)**	28 $\pm$ 10	27 $\pm$ 7	0.544
**Transpulmonary gradient**	8 (7-13)	11 (9-15)	0.067

Supplementary Table 2: Cardiopulmonary exercise and invasive pressure measurements of patients with and without severe MR before LVAD implantation; LVAD = Left ventricular assist device; MR = mitral regurgitation; VO_2 = Volume of Oxygen; METS = Metabolic equivalent of tasks; O_2 = Oxygen; HR = Heart rate; VE/VCO_2 = Minute ventilation/carbon dioxide production; PA = Pulmonary artery; PCWP = Pulmonary capillary wedge pressure;

Sharma H, *et al. Open Heart* 2023; 10:e002240. doi: 10.1136/openhrt-2022-002240

Supplementary Table 3: LVAD Procedural characteristics

	Severe MR (N = 29)	Non-severe MR (N = 98)	P value
**INTERMACS profiles:**			
**INTERMACS 1**	2 (7%)	8 (8%)	0.824
**INTERMACS 2**	10 (34%)	24 (24%)	0.286
**INTERMACS 3**	17 (59%)	66 (67%)	0.386
**Ventilated pre-operatively**	2 (7%)	7 (7%)	0.974
**Pre-LVAD mechanical circulatory support**			
- **IABP**	3 (10%)	9 (9%)	0.826
- **ECMO**	3 (10%)	4 (4%)	0.790
- **Impella**	3 (10%)	5 (5%)	0.307
**HeartMate II**	11 (38%)	31 (32%)	0.527
**HeartMate III**	18 (62%)	67 (68%)	0.527
**CPB time (mins)**	94 (76-117)	89 (78-114)	0.683
**Median ITU stay (days)**	9 (5-19)	8 (5-18)	0.955
**RVAD implantation**	4 (14%)	16 (16%)	0.742

Supplementary Table 3: LVAD procedural characteristics of patients with and without severe MR pre-LVAD; INTERMACS = International registry for mechanically assisted circulatory support; MR = mitral regurgitation; IABP = Intraaortic balloon pump; ECMO = Extra corporeal membrane oxygenation; CPB = Cardiopulmonary bypass; ITU = Intensive care unit; RVAD = Right ventricular assist device

Supplementary Table 4: Clinical outcomes following LVAD implantation

	BASELINE SEVERE MR (N=24)	BASELINE NON-SEVERE MR (N=91)	P VALUE
**HF HOSPITALISATION**			
NUMBER OF PATIENTS	6 (25%)	21 (23%)	0.843
MEAN DAYS POST-LVAD (TO FIRST EVENT)	214 (87-296)	454 (154-628)	0.122
**ALL MORTALITY**			
NUMBER OF PATIENTS	6 (25%)	34 (37%)	0.258
MEAN DAYS POST-LVAD (TO FIRST EVENT)	481 (200-556)	227 (90-940)	0.748
**HEART TRANSPLANTATION**			
NUMBER OF PATIENTS	6/29 (21%)	8/98 (8%)	0.044 **
MEDIAN DAYS POST-LVAD	233 (71-361)	897 (479-1725)	0.013 **
**DEFIBRILLATOR DEVICE**			
NUMBER OF PATIENTS	12 (50%)	42 (46%)	0.737

Supplementary Table 4: Comparison of clinical outcomes amongst patients with severe and non-severe MR pre-LVAD from implant to latest follow up. HF = Heart Failure; LVAD = Left Ventricular Assist Device

Sharma H, *et al. Open Heart* 2023; 10:e002240. doi: 10.1136/openhrt-2022-002240

Supplementary Table 5: LVAD settings and complications in patients with and without significant residual MR

	Significant Residual MR (N=15)	No significant residual MR (N=80)	P Value
**Indexed VAD flow (L/min/m²)**	2.8 ± 0.5	2.7 ± 0.5	0.479
**Operating speed (RPM)**			
HM II	9200 ± 566	8973 ± 1342	0.522
HM III	5750 ± 586	5615 ± 386	0.259
**Complications**	2 (13%)<br>- Pump thrombus n=1<br>- Outflow graft obstruction n=1	16 (20%)<br>- Sepsis n=6<br>- Stroke/TIA n=5<br>- Pump thrombus n=3<br>- Outflow graft obstruction n=1<br>- Device failure n=1	0.545
**Requirement of RVAD**	3 (20%)	11 (14%)	0.531

Supplementary Table 5: LVAD settings in patients with and without significant residual MR. LVAD = Left ventricular assist device; VAD = Ventricular assist device; MR = Mitral regurgitation; RPM = Rotations per minute; HM = HeartMate; TIA = Transient ischaemic attack

Supplementary Table 6: Comparison of residual MR in patients with and without baseline mild MR

	Inclusion of patients with baseline mild MR		P Value	Exclusion of patients with baseline mild MR		P value
	**Significant residual MR (N=15)**	**No significant residual MR (N=94)**		**Significant residual MR (N=14)**	**No significant residual MR (N=72)**	
**HF hospitalisation**	5 (33%)	21 (22%)	0.354	4 (29%)	17 (24%)	0.693
**All-cause mortality**	8 (53%)	28 (30%)	0.072	8 (57%)	20 (28%)	0.032 **

Supplementary Table 6: Comparison of residual MR in patients with and without baseline mild MR. This analysis was conducted to examine whether patients with residual MR had worse outcomes and if so, prediction at baseline would allow high-risk patients to undergo mitral valve repair at the time of LVAD implantation. Previous studies have included patients with mild MR at baseline, but such patients would not be candidates for mitral valve repair. Therefore this analysis was conducted to compare the effect of this subgroup on outcomes. Patients who underwent cardiac transplantation before the clinical end-points above were excluded (group with baseline mild MR included n=11; group with baseline mild MR excluded n=9).

Sharma H, *et al. Open Heart* 2023; 10:e002240. doi: 10.1136/openhrt-2022-002240
